# A draft transcriptome of a parasite *Neocamacolaimus parasiticus* (Camacolaimidae, Plectida)

**DOI:** 10.21307/jofnem-2021-040

**Published:** 2021-04-01

**Authors:** Mohammed Ahmed, Funmilola Adedidran, Oleksandr Holovachov

**Affiliations:** Department of Zoology, Swedish Museum of Natural History, SE-104 05, Stockholm, Sweden; Department of Zoology, Stockholm University, SE-106 91, Stockholm, Sweden.

**Keywords:** Transcriptomics, Marine, Parasitism, Plectida, Sweden

## Abstract

Camacolaimidae is a clade of nematodes that include both free-living epistrate feeding forms and parasites of marine protozoans and invertebrates. *Neocamacolaimus parasiticus* is a parasite of marine polychaete worms. Given its phylogenetic affinities to free-living species, *Neocamacolaimus* can be a reference for research of the origin of parasitism in an aquatic environment. Here, we present a draft transcriptome obtained from a single post-parasitic juvenile individual of this species. The final assembly consists of 19,180 protein coding sequences (including isoforms) with the following BUSCO scores for Nematoda: 65.38% complete, 9.06% partial, and 25.56% missing, and for Metazoa: 79.45% complete, 3.17% partial, and 17.38% missing.

The nematode family Camacolaimidae from the order Plectida is a relatively small group of marine nematodes (Holovachov, 2014) long thought to be free-living selective deposit feeders and epistrate feeders (Wieser, 1953). Recent studies, however, revealed that some members of this clade are intracellular parasites of foraminiferans (Hope and Tchesunov, 1999; Holovachov, 2015; Miljutin and Miljutina, 2016; Tchesunov et al., 2000) and internal parasites of polychaetes (Tchesunov, 2009), including the recently described *Neocamacolaimus parasiticus* Holovachov and Boström, 2014 (Fig. 1). Originally found parasitizing a benthic polychaete *Sphaerosyllis* cf. *hystrix* collected near Bonden island west of Grundsund in the Lysekil Municipality of Sweden (Holovachov and Boström, 2014), the species was subsequently found in several other localities in Skagerrak. These are the island of Hållö south-west of Smögen, localities west of Fjällbacka and around Koster islands, always in the coarse shell gravel characterized by the presence of lancelets (so-called amphioxus sand). Although relatively common in shell gravel, the species remains known only from infective and post-parasitic juveniles and males, while females were never found, despite multiple attempts over several years.

**Figure 1:**
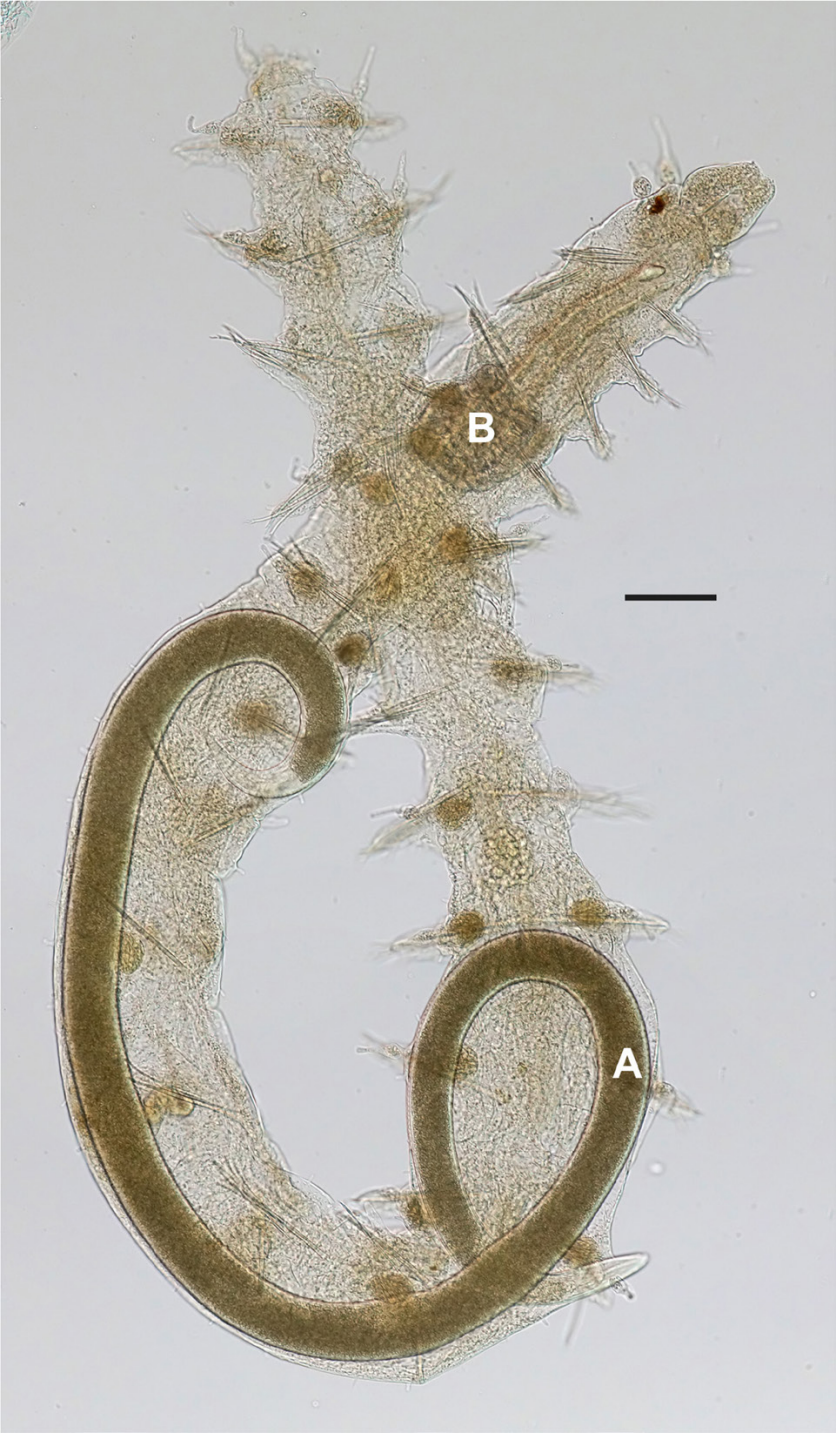
Parasitic juvenile of *Neocamacolaimus parasiticus* Holovachov and Boström, 2014 (A) emerging from the polychaete host *Sphaerosyllis* cf. *hystrix* (B). Scale bar 50 µm.

Within the order Plectida itself, the symbiotic lifestyle appears in several other lineages in addition to Camacolaimidae. These include endosymbionts of annelids from the family Ochridiidae, parasites of annelids from the family Creagrocercidae (Holovachov, 2014) and parasites of marine invertebrates from the family Benthimermithidae (Leduc and Zhao, 2019). At the same time, Plectida are a sister clade to Rhabditida (Secernentea in older literature), which includes the majority of currently known nematode diversity, and the majority of animal and plant parasitic species, as well as model species (Smythe et al., 2019). As such, plectids in general, and camacolaimids in particular represent a potentially interesting satellite model for the study of the origin of animal parasitism in the aquatic environment.

Sediment samples were collected near Koster islands with a bottom dredge and processed in the lab. Nematodes were extracted from the Amphioxus sand using a decanting and sieving method (Higgins and Thiel, 1988). Post-parasitic juveniles were fixed immediately in RNAlater solution and stored at −20°C. Total RNA was extracted from a single juvenile using the Ambion RNAqueous Micro Kit following the manufacturer’s protocol. Subsequent library preparation and cDNA synthesis was performed using the Takara Bio SMART-Seq HT Kit following manufacturer’s instructions. Resulting double-stranded cDNA was purified using the AMPure XP for PCR purification kit. Final library preparation and transcriptome sequencing were performed at Macrogen Europe B.V (Amsterdam, the Netherlands) using the Illumina Nextera DNA XT library preparation protocol and an Illumina HiSeq X sequencing technology (150PE). Raw reads were first filtered using fastp (Chen et al., 2018) with default settings (Q-score ≥ 15), and then assembled de novo using Trinity v2.9.1 (Grabherr et al., 2011) installation on the Uppsala Multidisciplinary Center for Advanced Computational Science (UPPMAX, www.uppmax.uu.se/) with default settings and in-silico normalization of reads, resulting in 66,328 contigs (73,030,971 bases). BUSCO scores for the initial assembly were: 70.16% complete, 9.06% partial, and 20.77% missing out of 982 reference genes for Nematoda and 83.95% complete, 5.42% partial, and 10.63% missing out of 978 reference genes for Metazoa (Waterhouse et al., 2018).

Assembled transcripts were filtered using Transrate (Smith-Unna et al., 2016) with default settings. The process inspected contig sequences and mapped filtered reads to the contigs and inspected the alignments. The goal was to remove chimeras, and poorly supported contigs from the assembly. Retained contigs (*n* = 42,787) were used to predict protein coding regions with TransDecoder 5.5.0 (transdecoder.github.io) against the most recent version of UniProt SwissProt database and PFAM (The UniProt Consortium, 2018). In order to remove potential contaminants, 30,435 protein sequences were compared against the custom database composed of protein sequences from six representative nematode genomes (*Acrobeloides nanus* PRJEB26554, *Brugia malayi* PRJNA10729, *Caenorhabditis elegans* PRJNA13758, *Plectus sambesii* PRJNA390260, *Pristionchus pacificus* PRJNA12644, *Trichuris muris* PRJEB126) downloaded from WormBase (Harris et al., 2020) using DIAMOND (Buchfink et al., 2015). All proteins not meeting the required threshold (*E*-value < 10-5, see Phillips et al., 2017 and Huang et al., 2019) were removed, resulting in 23,941 remaining protein sequences. Furthermore, CroCo (Simion et al., 2018) was used to detect and remove cross-species contamination in the dataset, by comparing the expression levels of different transcripts in samples prepared and sequenced together. However, instead of using the initial assemblies, we only checked for cross-contamination in the contigs that corresponded to filtered protein-coding sequences. CroCo removed 728 sequences considered as overexposed, contaminants, dubious and low coverage, leaving 23,213 ‘clean’ protein sequences. At the end, seqkit (Shen et al., 2016) was used to remove all duplicate sequences and all sequences shorter than 50 amino acids in length.

The final assembly consists of 19,180 protein-coding sequences (including isoforms) with the following BUSCO scores for Nematoda: 65.38% complete, 9.06% partial, and 25.56% missing (out of 982 reference genes), and for Metazoa: 79.45% complete, 3.17% partial and 17.38% missing (out of 978 reference genes).
